# Heterostructured Intermetallic Janus System with Atomic Precision

**DOI:** 10.1021/acscentsci.2c01010

**Published:** 2022-09-12

**Authors:** Xi Kang, Manzhou Zhu

**Affiliations:** Department of Chemistry and Centre for Atomic Engineering of Advanced Materials, Key Laboratory of Structure and Functional Regulation of Hybrid Materials of Ministry of Education, Anhui Province Key Laboratory of Chemistry for Inorganic/Organic Hybrid Functionalized Materials, Anhui University, Hefei, Anhui 230601, China

Janus nanoarchitectures, an emerging class of nanostructures, named after the Roman god with two faces, are a fascinating class of nanomaterials with promising applications in various areas, including catalysis, optical imaging, and so on. Although matter with structural and chemical homogeneity tends to display high stability owing to the low entropy, its heterogeneous counterpart is also attractive because of the high reactivity arising therefrom. To date, several bimetallic Janus nanocrystals have been fabricated; however, the atomic-level investigation of their structure–property correlations remains highly challenging due to two intrinsic characteristics: ununiform sizes and imprecise surface chemistry. In this issue of *ACS Central Science*, Shuang-Quan Zang and co-workers have structurally resolved Janus nanoarchitectures at the atomic level and mapped out their interfacial linkages and the synergistic effect.^1^

**Figure 1:**
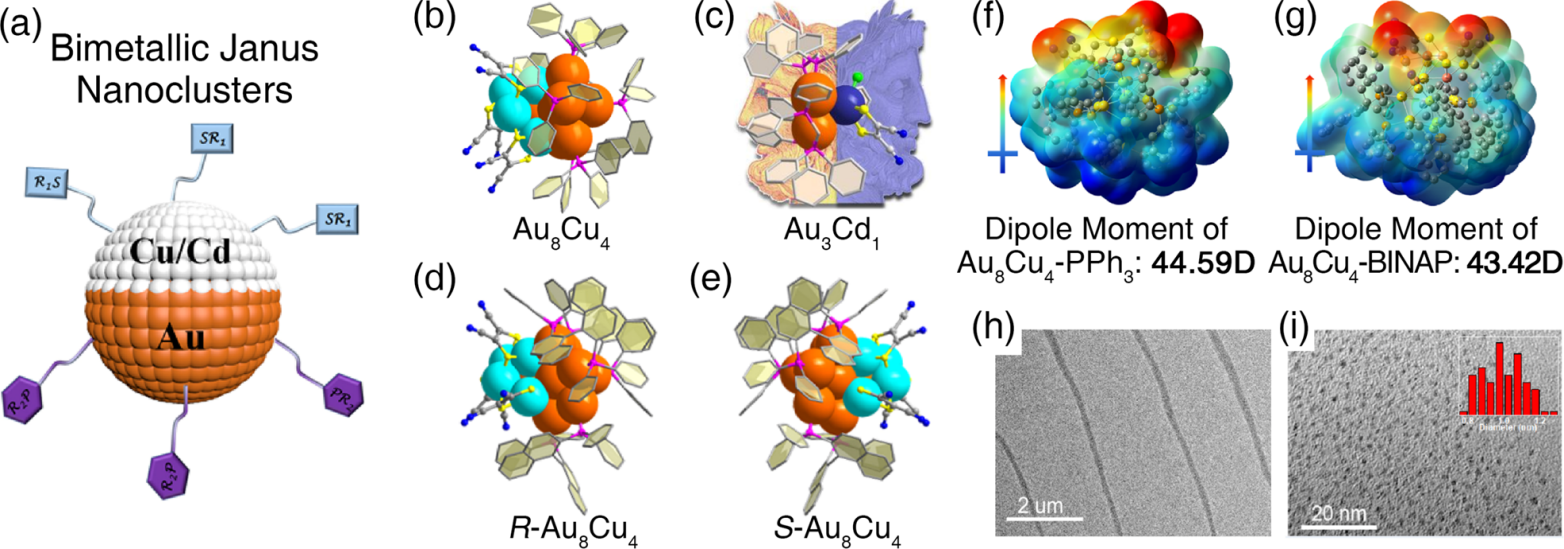
(a) Scheme illustration of the bimetallic Janus structures of the obtained nanoclusters. (b, c) Structures of the racemate Au_8_Cu_4_ and Au_3_Cd_1_ nanoclusters. (d, e) Structures of *R*/*S*-Au_8_Cu_4_ enantiomers. Color legends: orange sphere, Au; turquoise sphere, Cu; indigo sphere, Cd; pink sphere, P; yellow sphere, S; blue sphere, N; green sphere, Cl; gray sphere, C. Hydrogen atoms are omitted for clarity. (f, g) Directions and values of the dipole moment of Au_8_Cu_4_ nanoclusters. (h, i) TEM images of the one-dimensional nanowires made from Au_8_Cu_4_ nanocluster molecules. Reproduced with permission from ref (1). Copyright 2022 The Authors. Published by American Chemical Society.

In this work, four Janus nanoclusters, including racemate Au_8_Cu_4_, *R*/*S*-Au_8_Cu_4_ enantiomers, and racemate Au_3_Cd_1_, costabilized by thiol and phosphine ligands, were controllably synthesized and structurally determined, serving as research templates to resolve fundamental issues of asymmetric bimetallic Janus nanocrystals. Structurally, these four alloy nanoclusters perfectly reflected the two-face characterization of Janus architectures (Figure 1a–e): the gold sides are stabilized by phosphine ligands (i.e., PPh_3_, BINAP, and DPPM), whereas the transition metal copper/cadmium sides are anchored by thiol ligands (i.e., MNT). In addition, the six achiral PPh_3_ ligands in racemate Au_8_Cu_4_ could be substituted by diphosphine BINAP ligands, and the postmodified synthesized *R*/*S*-Au_8_Cu_4_ nanocluster enantiomers exhibited obvious chiroptical properties. By analyzing the density functional theory calculation results of the Au_8_Cu_4_ nanocluster, the authors demonstrated that the Au–Cu interaction may play a critical role in forming the Janus nanocluster, and the lattice adaptability from the protecting thiol/phosphine ligands induced asymmetric growth. In addition, the dipolar distribution of Au/Cu bicomponents in Au_8_Cu_4_ led to a maximum dipole moment up to 45 D (Figure 1f,g), which further drove the self-assembly of Au_8_Cu_4_ nanocluster molecules into one-dimensional nanowires (Figure 1h,i).

**Figure 2:**
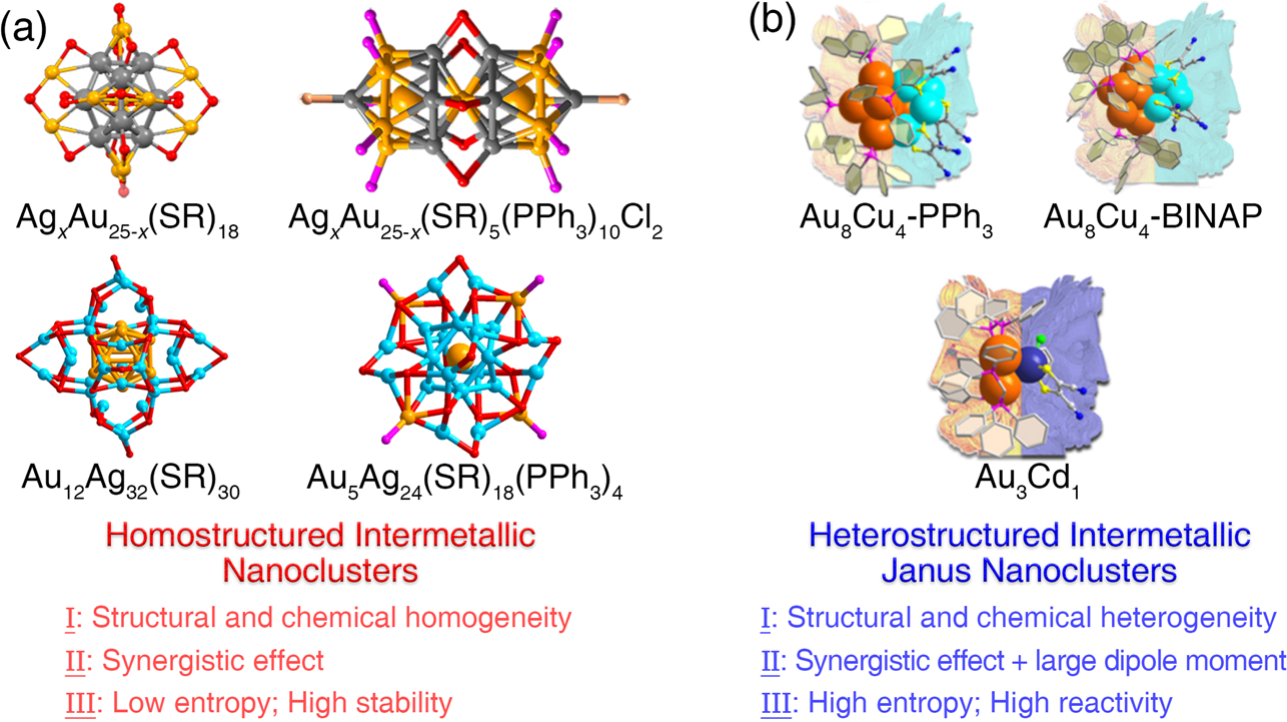
(a) Structural and chemical homogeneity in several alloy nanoclusters with high stability, such as Ag_*x*_Au_25–*x*_(SR)_18_, Ag_*x*_Au_25–*x*_(SR)_5_(PPh_3_)_10_Cl_2_, Au_12_Ag_32_(SR)_30_, and Au_5_Ag_24_(SR)_18_(PPh_3_)_4_ nanoclusters. (b) Structural and chemical heterogeneity in several Janus nanoclusters with high reactivity, such as Au_8_Cu_4_ and Au_3_Cd_1_ nanoclusters. Reproduced with permission from ref (1). Copyright 2022 The Authors. Published by American Chemical Society.

Through the continuous accumulation of synthetic experience and advances in analytical methods, metal nanoclusters can now easily be tailored to desired composition and morphology. The concept of alloying has been extensively exploited in dictating the geometric/electronic structures and customizing the chemical/physical properties of metal nanoclusters.^2^ In most alloying cases, the active alloying sites tend to have more equal distributions in metallic skeletons of metal nanoclusters (Figure 2a). For example, the incorporated Ag heteroatoms prefer to occupy the icosahedral kernel surface with a uniform pattern in Ag_*x*_Au_25–*x*_(SR)_18_ nanoclusters;^3^ the architecture of the rodlike Ag_*x*_Au_25–*x*_(SR)_5_(PPh_3_)_10_Cl_2_ nanocluster perfectly follows a symmetrical alloying mode along the central plane;^4^ the alloyed Au_12_Ag_32_(SR)_30_ nanocluster follows a Au_12_ core@Ag_32_ shell architecture of the structural aesthetic;^5^ the five Au heteroatoms in the tetrametallic Au_5_Ag_24_(SR)_18_(PPh_3_)_4_ nanocluster are evenly arranged into the innermost kernel or onto the outermost vertexes of the cluster skeleton.^6^ The equal distribution of heterometals in these commonly researched alloy nanoclusters is reasonable because the structural and chemical homogeneity should reduce the entropy value of a cluster system and produce an alloy nanocluster with high stability. Such a tendency is in line with the expressions of “*life feeds on negative entropy*” and “*survival of the fittest*”.^7,8^

Janus nanoclusters, on the other hand, exhibit structural and chemical heterogeneity. The asymmetrical structures endow these Janus nanoclusters with distinctively chemical–physical properties owing to the emerging large dipole moments, in addition to the synergistic effect between different metal components. In addition, the high reactivity of Janus nanomaterials originating from their dynamically unfavorable characterization makes them well-suited as optical devices or nanocatalysts.^9^ In this work, the Janus Au_8_Cu_4_ nanoclusters displayed high surface energy and were self-assembled into cluster-based nanowires with the driving force of their inherent bipolar phase and intercluster dipole interactions. In addition, the photocurrent response properties of such Janus nanoclusters were excellent, manifesting good photogenerated electron/hole pair generation and separation efficiencies.^1^

However, the bottom-up synthesis of Janus nanoclusters remains highly challenging since they are dynamically unfavorable and require a delicate interplay balance between entropy and enthalpy.^10^ Shuang-Quan Zang and co-workers prepared such Janus nanoclusters by using mixed ligands with different metallic affinities and electronegativities of the substituents.^1^ We envision that such a controllable method (i.e., mixed ligands stabilizing noble/transition metals) will allow for the efficient fabrication of more atomically precise Janus nanostructures in the near future.

In summary, the four reported Au–Cu or Au–Cd Janus nanoclusters reported by Shuang-Quan Zang and co-workers resolved several fundamental issues at the atomic level, including the interfacial linkages and the synergistic effects in Janus nanoarchitectures. The new findings provide atomic-level clues for understanding the heterogeneous architectures of Janus nanomaterials. In addition, such new findings will hopefully pave the way for the future fabrication of heterostructured intermetallic Janus systems for several downstream applications in optics and catalysis.
